# Comparative Evaluation of Deep Learning Models for Diagnosis of Helminth Infections

**DOI:** 10.3390/jpm15030121

**Published:** 2025-03-20

**Authors:** Omid Mirzaei, Ahmet Ilhan, Emrah Guler, Kaya Suer, Boran Sekeroglu

**Affiliations:** 1 Department of Biomedical Engineering, Faculty of Engineering, Near East University, Nicosia 99138, TRNC, Mersin 10, Turkey; omid.mirzaei@neu.edu.tr; 2 Department of Computer Engineering, Faculty of Engineering, Near East University, Nicosia 99138, TRNC, Mersin 10, Turkey; ahmet.ilhan@neu.edu.tr; 3 Department of Molecular Biology and Genetics, Faculty of Arts and Sciences, European University of Lefke, Lefke 99010, TRNC, Mersin 10, Turkey; eguler@eul.edu.tr; 4 Department of Infectious Diseases and Clinical Microbiology, Faculty of Medicine, Near East University, Nicosia 99138, TRNC, Mersin 10, Turkey; kaya.suer@neu.edu.tr; 5 Department of Information Systems Engineering, Faculty of Engineering, Near East University, Nicosia 99138, TRNC, Mersin 10, Turkey; boran.sekeroglu@neu.edu.tr

**Keywords:** helminth infections, *Ascaris lumbricoides*, *Taenia saginata*, deep learning, microscopic image classification

## Abstract

(1) **Background**: Helminth infections are a widespread global health concern, with Ascaris and taeniasis representing two of the most prevalent infestations. Traditional diagnostic methods, such as egg-based microscopy, are fraught with challenges, including subjectivity and low throughput, often leading to misdiagnosis. This study evaluates the efficacy of advanced deep learning models in accurately classifying *Ascaris lumbricoides* and *Taenia saginata* eggs from microscopic images, proposing a technologically enhanced approach for diagnostics in clinical settings. (2) **Methods**: Three state-of-the-art deep learning models, ConvNeXt Tiny, EfficientNet V2 S, and MobileNet V3 S, are considered. A diverse dataset comprising images of Ascaris, Taenia, and uninfected eggs was utilized for training and validating these models by performing multiclass experiments. (3) **Results**: All models demonstrated high classificatory accuracy, with ConvNeXt Tiny achieving an F1-score of 98.6%, followed by EfficientNet V2 S at 97.5% and MobileNet V3 S at 98.2% in the experiments. These results prove the potential of deep learning in streamlining and improving the diagnostic process for helminthic infections. The application of deep learning models such as ConvNeXt Tiny, EfficientNet V2 S, and MobileNet V3 S shows promise for efficient and accurate helminth egg classification, potentially significantly enhancing the diagnostic workflow. (4) **Conclusion**: The study demonstrates the feasibility of leveraging advanced computational techniques in parasitology and points towards a future where rapid, objective, and reliable diagnostics are standard.

## 1. Introduction

Helminth infections are commonly seen worldwide and continue to be a public health problem [1,2]. Helminth infections are more frequently encountered in underdeveloped regions with tropical climates. However, increased transportation opportunities due to developing technology, mass migration, asylum, tourism, and war facilitate the spread of helminth infections to regions where they are not seen [3]. Helminths are frequently transmitted to humans through various routes, including hands, food, and water contaminated with human and animal feces or soil [2].

In general, two groups of helminths are important for human health. These are called nemathelminths (roundworms or nematodes) and plathelminths (flatworms, trematodes, and cestodes) [1,2]. In particular, soil-transmitted helminth (STH) infections are among the most common infections, infecting approximately 24% of the world’s population with 1.5 billion cases. These infections affect poor communities in tropical and subtropical regions where access to clean water, sanitation, and hygiene is poor and/or lacking, with the highest prevalence reported in sub-Saharan Africa, China, South America, and Asia [4]. Health impacts include anemia, retarded physical growth, reduced work capacity, and complications during pregnancy. People with mild infections usually have no symptoms, while more severe infections can cause various symptoms, including diarrhea, abdominal pain, weakness, intestinal bleeding, loss of appetite, decreased food intake, and reduced physical fitness [5].

Four parasites are responsible for STH infections worldwide: *Ascaris lumbricoides*, *Trichuris trichiura*, Hookworm, and *Strongyloides stercoralis* [6]. *Ascaris lumbricoides* is one of the most common helminths in the gastrointestinal tract, and Ascariasis causes infection [7,8]. It is estimated that approximately 807 million–1.2 billion people in the world are infected with *Ascaris lumbricoides* [8]. Ascariasis is caused by ingesting embryonated eggs through contaminated food, water, and soil; this parasite lives in the small intestine. Females produce eggs, which are released in the feces of infected individuals [6].

The diagnosis of Ascariasis is primarily based on microscopy, looking for *Ascaris lumbricoides* eggs in stool samples of infected individuals. However, seeing the eggs under stool microscopy is challenging, and specialized laboratory personnel are needed. The parasite’s life cycle has three different forms of eggs: infertile, fertilized with a sheath, and fertilized without a sheath. Fertilized eggs are oval-shaped and 40 × 60 μm in size. Unfertilized eggs, on the other hand, are larger and longer (60 × 90 μm) than fertilized eggs, with thinner shells and granules of various sizes [9,10]. This polymorphism in the egg causes the egg to have the potential to be confused with non-parasitic substances (artifacts). Therefore, it is important to distinguish between artifacts (pollen, plant cells, etc.) and parasite eggs to avoid misdiagnosis. To distinguish between parasitic and non-parasitic elements, laboratory professionals need to be familiar with the complex characteristics of eggs (e.g., size, shape, shell structure, and internal characteristics of AL eggs). It is recognized that the microscope method commonly used in routine laboratories increases the likelihood of such misdiagnosis [10,11].

The diagnosis of patients with tenia is based on direct microscopy of eggs in the stool. Although this diagnostic method is relatively easy to implement in resource-limited areas, a major drawback is the sensitivity of the microscopy method due to the intermittent nature of egg shedding, with published sensitivity estimates ranging from 3.9% to 52.5% [12]. The Taenia eggs are indistinguishable from each other and other members of the Taeniidae family. Eggs are 30–35 μm in diameter and radially striated. The inner oncosphere contains six break-resistant hooks [13].

The abovementioned challenges and limitations caused researchers to seek more accurate diagnostic and detection tools for *Ascaris lumbricoides* and *Taenia saginata*. Artificial Intelligence (AI) has become a significant and powerful tool to assist experts in detecting or diagnosing diseases [14,15,16,17] due to its efficacy in extracting different levels of features and establishing non-linear relationships between the features and diseases. In this era, microbiologists have an opportunity to collect vast amounts of biological data, where analyzing data requires advanced machine learning methods, particularly deep learning. Even though traditional machine learning methods enhanced microbiology [18] in identifying potential vaccine and drug targets, diagnosing microorganisms that cause infectious diseases, predicting epidemics, and studying microbial interactions, they are no longer effective in diagnosing and detecting diseases or parasites due to the huge amount of images. Deep learning (DL) often performs better in terms of accuracy and speed compared to traditional methods, and the adoption of these innovative approaches by microbiologists will lead to new applications for their field of study, develop new perspectives, and provide new insights [19].

For that reason, several studies were performed to detect or classify helminth infections using deep learning approaches. Even though these studies achieved reasonable accuracy rates, considering a single deep learning model created limitations for researchers to analyze the efficacy of different models on recognizing helminth infections. On the other hand, a few studies considered multiple models for comparing new-generation pre-trained models’ performances. Although these studies provided valuable information for first-generation deep learning models, they could not provide the performance analysis of new-generation deep learning models, which are more accurate.

This study aims to demonstrate the efficacy of the new-generation deep learning models by performing a comparative analysis in classifying helminth infections, *Ascaris lumbricoides*, *Taenia saginata*, and uninfected patients. Three models, EfficienNetV2 S, ConvNeXt Tiny, and MobileNetV3, are implemented, and the results are analyzed in detail. Additionally, statistical assessment is performed to demonstrate the reliability of the results obtained by the models.

The rest of the paper is organized as follows: Section 2 summarizes the recent related studies, and Section 3 introduces the primary dataset, considered models, and experimental design of the study. Section 4 presents the results and Section 5 discusses the obtained results. Finally, the conclusion is presented in Section 6.

## 2. Literature Review

He et al. [20] collected eggs of *A. lumbricoides*, *Trichuris trichiura*, *Enterobius vermicularis*, *Ancylostoma duodenale*, *Schistosoma japonicum*, *Paragonimus westermani*, *Fasciolopsis buski*, *Clonorchis sinensis*, and *Taenia* spp. and prepared them as single species and mixed egg smears. Eggs were photographed under a light microscope and analyzed with the YOLO v4 model. As a result of the analyses, the accuracy rate for *Clonorchis sinensis* and *Schistosoma japonicum* was 100%, while *Enterobius vermicularis* (89.31%), *Fasciolopsis buski* (88.00%), and *Trichuris trichiura* (84.85%) were also high. In addition, accuracy rates for mixed helminth eggs were obtained for Group 1 (98.10%, 95.61%), Group 2 (94.86%, 93.28%, 91.43%), and Group 3 (93.34%, 75.00%). The model provided advantages in terms of sensitivity and specificity for certain parasite types but struggles in mixed infections and complex diagnostic scenarios.

In the research by Butploy et al. [9], a deep learning method for recognizing *A. lumbricoides* egg images was proposed, and a prototype tool for parasite egg detection in medical diagnosis was developed. The challenge is recognizing three different egg types of *A. lumbricoides* with optimal deep-learning architecture. The results showed that the classification accuracy of parasite eggs reaches up to 93.33%. The efficacy of the proposed model showed that the time-consuming process of parasite egg classification could be reduced by implementing artificial intelligence and deep learning models.

Martinez Pastor GG et al. [21] focused on a Convolutional Neural Network (CNN) model. Through this approach, the training, testing, and validation phases of the CNN model for detecting and recognizing *A. lumbricoides* parasite eggs were performed. The results showed that the proposed CNN model, in combination with image preprocessing, yielded extremely positive results in parasite egg identification. Moreover, very satisfactory values were obtained in the model testing and validation phases, indicating its effectiveness and accuracy in diagnosing the presence of parasites. The authors concluded that comprehensive experiments with different parasite egg datasets are required to develop a system to assist experts in identifying eggs.

Muthulakshm et al. [22] developed an automated system to accurately classify parasite egg types in microscopic images using the ability of compression alert layers to learn general knowledge from the input. The proposed system uses ResNet50 and ResNet101 for analysis and the features extracted by the Squeeze Excitation (SE) layers. The extracted features are then given as input to a support vector classifier. The study provides a systematic evaluation of the features extracted from ResNet50+SE and ResNet101+SE. The results from the evaluation show the effectiveness of ResNet50+SE in classifying parasite egg types in microscopic images with 94% accuracy. This study demonstrated that pretrained deep learning models could effectively extract the features and could provide a high-accuracy classification of the eggs using other classifiers.

Ali Mansour Abdelmula et al. [23] evaluated alternative diagnostic strategies for recognizing amastigotes of cutaneous leishmaniasis parasites at different stages using fractional neural networks (CNNs). Among the CNN models, pre-trained deep learning models such as EfficientNetB0, DenseNet201, ResNet101, MobileNetv2, and Xception are used. Following a thorough evaluation and comparison of various models, DenseNet-201 turned out to be the most appropriate choice. This model achieved an average accuracy of 0.9914, with outstanding results for the main classification performance metrics such as sensitivity, specificity, positive predicted value, negative predicted value, F1-score, Matthew’s correlation coefficient, and Cohen’s Kappa coefficient. This study demonstrated that deep learning models could provide a balanced combination of precision and sensitivity while exhibiting high classification capabilities.

## 3. Materials and Methods

### 3.1. Dataset

The stool samples containing *Ascaris lumbricoides* and *Taenia saginata* eggs used in our study belonged to patients diagnosed in the Near East University (NEU) Hospital Microbiology Laboratory and Manisa Celal Bayar University Parasitology Laboratory. Stool samples were collected from the patients with gastrointestinal complaints, and all samples were evaluated and diagnosed by infectious disease specialists and expert microbiologists. Stool samples in which *Ascaris lumbricoides* and *Taenia saginata* eggs were detected were stored at −80 °C until use.

**Figure 1:**
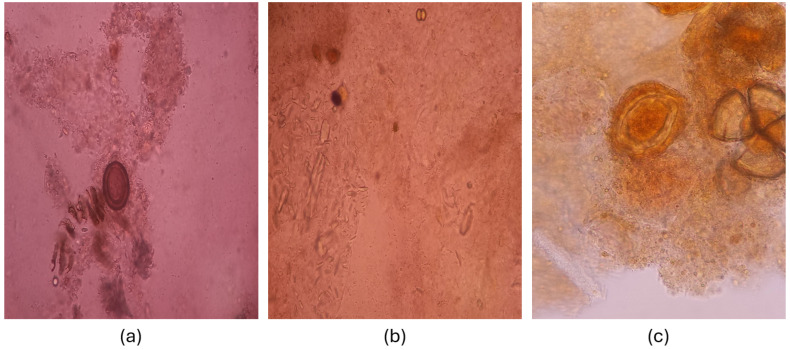
Sample dataset images (**a**) *Taenia saginata*. (**b**) Uninfected. (**c**) *Ascaris lumbricoides*.

All stool samples were prepared using Lugol solution and examined under a light microscope with a 40× objective to detect parasite eggs. Images are captured with a personal mobile phone using a microscope-mobile phone apparatus from the areas with parasite eggs. A total of 1324 infected images were obtained (884 for *Ascaris lumbricoides* and 440 images for *Taenia saginata* eggs), while 1003 images were captured for uninfected eggs to create a Helminth Egg Microscopy Image Collection (HEMIC) dataset. The basic characteristics of the HEMIC) dataset are provided in Table 1, and sample images are shown in Figure 1.

### 3.2. Deep Learning Models

This section provides a detailed overview of the deep learning models that have been considered for the classification task involving Ascaris and Taenia parasites.

#### 3.2.1. ConvNeXt

**Figure 2:**
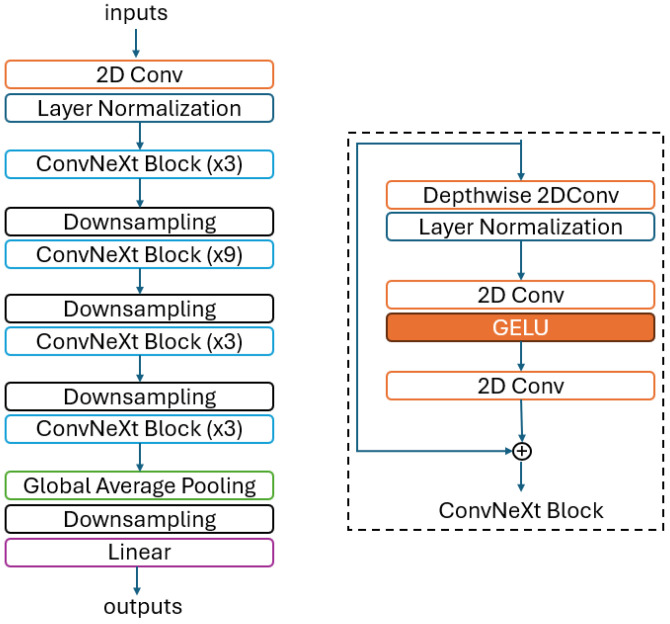
Basic block diagram of the ConvNeXt model.

ConvNeXt differentiates itself by leveraging established techniques and optimized convolutional neural networks (CNNs) to enhance performance without introducing novel architectural or methodological innovations. By adopting the Vision Transformer (ViT) training strategy, ConvNeXt achieves significant performance improvements compared to the baseline, setting a benchmark for subsequent experiments. This optimized approach demonstrates the ability of the ConvNeXt to enhance performance without sacrificing efficiency. ConvNeXt outperforms the Swin Transformer in rigorous testing, maintaining equivalent computational complexity while achieving faster inference speed and higher accuracy. This makes ConvNeXt a promising solution for image processing tasks, as it optimizes performance without compromising efficiency [24]. Figure 2 shows the basic block diagram of the ConvNeXt model with the ConvNeXt Block.

#### 3.2.2. EfficientNet

**Figure 3:**
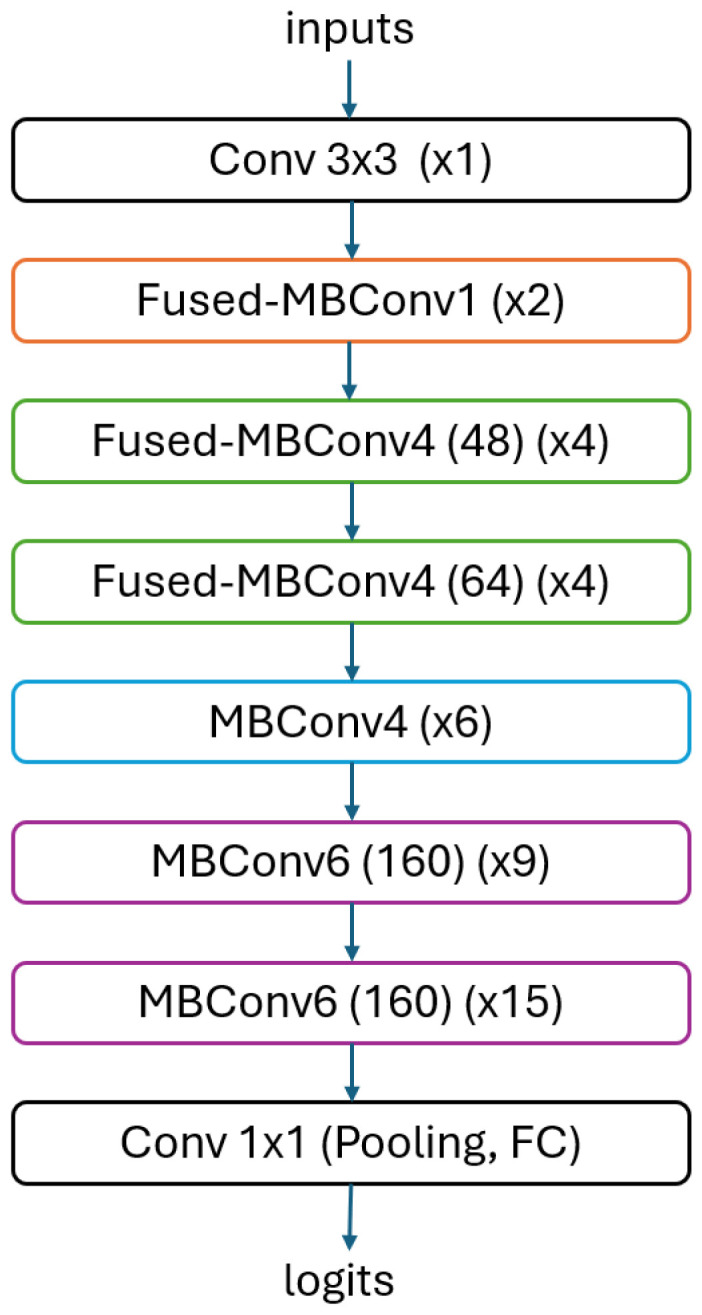
Basic block diagram of the EfficientNet V2 S model.

The composite scaling method proposes scaling the width, depth, and resolution of the network using fixed scaling coefficients. This approach, referred to as NAS, has led to the development of a novel base network known as EfficientNets, aimed at delivering enhanced compactness and swiftness in contrast to existing convolutional neural networks. In June 2021, the introduction of EfficientNetV2 marked a milestone by amalgamating NAS and scaling, thereby enhancing training speed and parameter efficiency. Researchers expanded the exploration scope by incorporating additional operations and introducing a progressive learning technique that dynamically regulates regularization and image dimensions. The EfficientNetV2 models provide approximately 50% faster training compared to its initial version. However, during the minimization of the training time, the recognition ability is improved for relatively small datasets with fused convolutional layers. These characteristics make EfficientNetV2 models more suitable for transfer learning. EfficientNetV2 has exhibited a substantially accelerated training pace and superior parameter efficiency when juxtaposed with previous methodologies such as ResNet-101 and ViT-L/16 on diverse datasets like ImageNet, CIFAR, Cars, and Flowers [25,26]. Figure 3 presents the basic layers of the EfficientNet V2 S model.

#### 3.2.3. MobileNet

**Figure 4:**
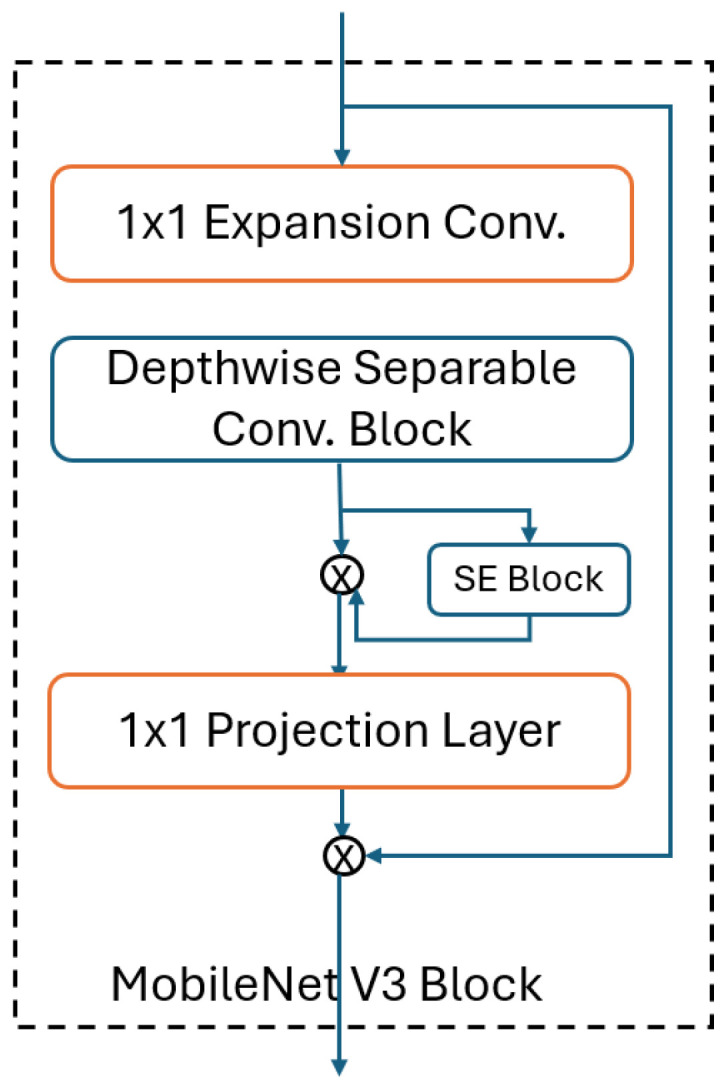
Basic block diagram of the MobileNet V3 Block.

MobileNet, a Convolutional Neural Network (CNN) architecture, utilizes separable depth-wise convolutions that comprise standard, depth-wise, and point-wise convolutions. This innovative design effectively reduces both computation and model size, optimizing efficiency. MobileNet’s structure incorporates a width multiplier parameter, uniformly decreasing network size at each layer. This enables MobileNet to be compact, has low latency, and is well-suited for computationally limited platforms while maintaining robust performance. The creators have introduced MobileNetV2, enhancing the network with inverted residual and linear bottleneck techniques. In MobileNetV3, a combination of depth-wise separable convolutions and inverted residual with linear bottleneck modules is utilized. Employing platform-aware Neural Architecture Search (NAS) and the NetAdapt algorithm, the authors explore network structures and determine the ideal number of filters per layer. Moreover, computationally expensive layers are redesigned, and the ReLU nonlinearity is replaced with the swish activation function. MobileNetV3 achieves higher accuracy and lower latency compared to MobileNetV2, establishing itself as the next generation of the MobileNet series. This advancement marks a significant milestone in the evolution of CNN architectures, offering increased efficiency and performance for a wide range of applications [27,28]. MobileNetV3 can be used in low-power environments, and the model has a relatively small size compared to the other pre-trained deep learning models, making it more suitable to be implemented in mobile devices. Figure 4 shows the basic MobileNet V3 block.

Due to the abovementioned efficacies of these models, we selected them to perform a comparative study in recognizing Helminth Infections.

### 3.3. Experimental Design

**Figure 5:**
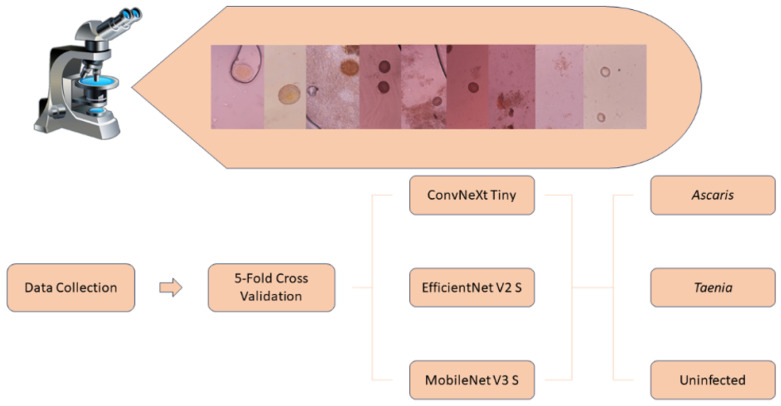
Block diagram of the phases of the study.

The experiments consisted of the multinomial classification of infections and uninfected samples. In these experiments, the ability to diagnose infection types and healthy samples is analyzed to provide a reference study for further research. Figure 5 demonstrates the general block diagram of this study by illustrating the experiments.

All experiments are performed using 5-fold cross-validation in order to obtain more accurate results and analyze the performance of the models. Deep learning models are trained using the Adam optimizer with a batch size of 32 and a learning rate of 0.0001. Each model is trained for 100 epochs to ensure comprehensive learning. The mean results of all folds are considered the final results for each model.

Images are resized during the training to satisfy the model’s input size. Image preprocessing techniques such as noise reduction were not applied to maintain the raw characteristics and ensure that the model trains the original data. Data augmentation is not considered to observe the efficacy of the models without additional data. The normalization of the images is performed using the internal normalization procedure of each model.

A personal computer with an i9-9th generation CPU, 32 GB of RAM, and an NVIDIA GeForce RTX 2080-Ti graphics processor (NVIDIA, Santa Clara, CA, USA) is used in all experiments.

### 3.4. Evaluation Metrics

The common evaluation metrics for the classification tasks are considered to analyze the results obtained by the models. We used recall, precision, and F1-Score metrics for the evaluation.

(1) $$\mathrm{Recall}=\frac{\mathrm{TP}}{\mathrm{TP}+\mathrm{FN}}$$

(2) $$\mathrm{Precision}=\frac{\mathrm{TP}}{\mathrm{TP}+\mathrm{FP}}$$

where TP and TN denote correctly classified positive and negative samples, while FP and FN represent misclassified positive and negative samples.

(3) $$F1\;\mathrm{Score}=\frac{2\times \mathrm{Precision}\times \mathrm{Recall}}{\mathrm{Precision}+\mathrm{Recall}}$$

## 4. Results

This section presents the results of all experiments in detail and provides the statistical analysis.

### 4.1. Experimental Results

**Figure 6:**
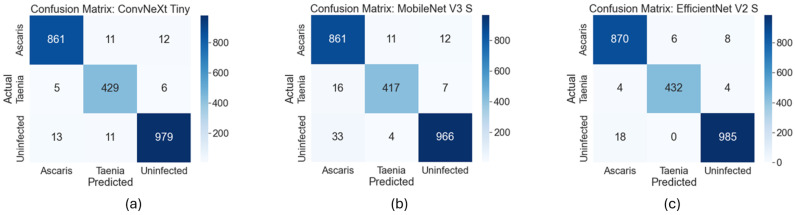
Confusion matrices (**a**) ConvNeXt Tiny. (**b**) MobileNet V3 S. (**c**) EfficientNet V2 S.

Three deep learning models were investigated for the classification of microscopic images into *Ascaris*, *Taenia*, and Uninfected. All models achieved reasonable results in distinguishing parasite type or uninfected samples. Even though the ConvNeXt Tiny model achieved the highest precision score for Ascaris, EfficientNet V2 S obtained superior results for other metrics and parasites. Performance measures for parasite classification using three different models with five-fold cross-validation are presented in Table 2, and the confusion matrices of each model are presented in Figure 6.

The results are also evaluated by calculating macro averages. Evaluation results showed that the EfficientNet V2 S model achieved superior results and outperformed other models. Table 3 presents the comparative macro-averaged results for the models in detail.

### 4.2. Statistical Assessment with ANOVA

ANOVA (Analysis of Variance) serves as a statistical method for examining machine learning algorithms by offering a structured approach to determine the significance of distinctions among diverse groups in terms of performance metrics. This analytical tool allows researchers and practitioners to gauge the variations in model outcomes based on various input parameters, architectures, or datasets. While ANOVA conventionally finds its application in classical experimental designs, its integration into the realm of machine learning facilitates the understanding of how different factors influence model performance. Consequently, ANOVA proves to be a valuable instrument for the assessment and interpretation of machine learning models [29].

In this study, one-way ANOVA is applied for each evaluation metric, considering all fold results of the models in multinomial experiments to provide information about whether there is a statistically significant difference in evaluation metrics between the models. The significance level (threshold) for the *p*-value is determined as 0.05. The *p*-values of the evaluation metrics obtained from the One-Way ANOVA test are presented in Table 4.

Table 4 indicates that the *p*-value for precision exceeds the specified significance level. This implies that all models demonstrate comparable success rates in making positive predictions. However, a noteworthy disparity is observed in the recall metric. Specifically, a significant difference is identified through the Tukey HSD (Honestly Significant Difference) post-hoc test [30] between the EfficientNet V2 S and MobileNet V3 S models. This suggests a substantial variation between these two models in predicting true positives, with no significant difference observed in this regard for the ConvNext Tiny model.

Additionally, significant differences are noted in the F1-Score metric. In particular, the Tukey HSD test highlights a significant dissimilarity between the EfficientNet V2 S and MobileNet V3 S models, indicating variations in both recall and F1-Score for these two models. The FWER (Family-Wise Error Rate) for the Tukey HSD test is determined as 0.05. Detailed results obtained from the Tukey HSD test are presented in Table 5 and Table 6.

## 5. Discussion

This study aimed to apply computer-aided diagnosis (CAD) techniques for classifying parasites on microscopic images. We analyzed three effective deep-learning models to classify the primary dataset. The results showed that the deep learning models could accurately distinguish the Ascaris, Taenia, and uninfected images.

EfficientNet V2 S achieved superior results since it is an effective model for small datasets due to the strong optimizations embedded into the model. The optimization of the EfficientNet V2 S includes strong regularization and feature extraction techniques that differ from other models, including stochastic depth, depthwise and pointwise convolutions that help the model generalize better in limited training data. Therefore, it outperformed ConvNeXt Tiny and MobileNet V3 models by generalizing the test samples more accurately. However, increasing the training samples might help ConvNeXt Tiny and MobileNet V3 models achieve higher or superior results.

Even though the EfficienNet V2 S model achieved superior results, all models showed outstanding performance by obtaining a score higher than 0.96 F1-Score in general recognition ability. Muthulakshm M et al. [22] achieved 94% accuracy by considering ResNet models, while Butploy et al. [9] obtained 93.3% accuracy. The results also showed the efficacy of the new generation deep learning models, which are optimized to extract more distinct features and provide efficient classification ability. However, the practical implementation of the studies requires varied datasets that include more infection images acquired from different tools and external validation to observe the clinical impact of the deep learning models.

Statistical tests, including ANOVA and subsequent Tukey HSD comparisons, show that precision does not vary significantly between models evaluated (*p*-value = 0.0559). In practical terms, this shows that all models perform similarly in correctly identifying positive examples that they label as positive. However, recall (*p*-value = 0.0101) and F1-score (*p*-value = 0.0005) results reveal statistically significant differences; this implies that at least one model performs better or worse than the others in terms of capturing true positives (recall) and balancing precision with recall (F1-score).

Post-hoc Tukey HSD tests provide further insight into specific models that exhibit significant differences. In particular, EfficientNet V2 S and MobileNet V3 S show a clear difference in both recall and F1 scores. Negative mean differences (e.g., −0.0218 for recall) indicate that MobileNet V3 S achieves a higher recall when using EfficientNet V2 S as the baseline. A similar trend is observed in the F1 score. This shows that MobileNet V3 S provides a better balance between precision and recall compared to EfficientNet V2 S. Other pairwise comparisons reveal no significant differences. This shows that the remaining models, including ConvNext Tiny, perform similarly on these metrics.

**Figure 7:**
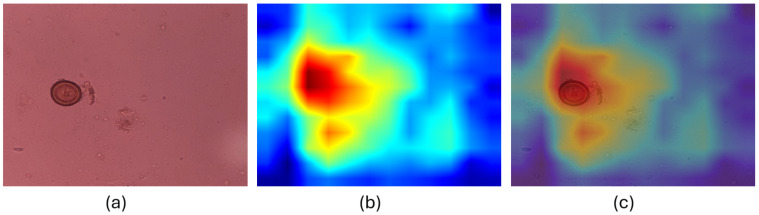
GradCAM of correctly classified *Taenia saginata* by EfficienNet V2 S Model (**a**) Original Image. (**b**) GradCAM. (**c**) Overlaid image.

**Figure 8:**
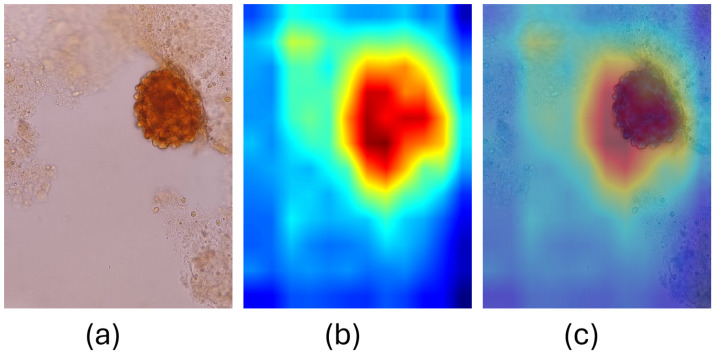
GradCAM of correctly classified *Ascaris lumbricoides* by EfficienNet V2 S Model (**a**) Original Image. (**b**) GradCAM. (**c**) Overlaid image.

Visualizing the regions that models focus on at the decision level is also important. We generated the Grad-CAMs of the superior model, EfficienNet V2 S, to demonstrate in which regions the model made decisions. Figure 7 and Figure 8 show the Grad-CAMs that the model is focused on the appropriate regions.

This study has some limitations. The study’s primary limitation is the number of images, whereas the higher number of training samples provides more information to the deep learning models to lead to practical implementation. The secondary limitation is not evaluating the trained models with the external, which is critical for the real classification ability of the models that provide clinical implementation. We aim to overcome these limitations by collecting more laboratory samples and collaborating with researchers worldwide. This will lead us to obtain varied and higher numbers of data. The other limitation of the study is that it does not employ any preprocessing techniques for the images, while noise removal or sharpening might increase the classification ability of the models. In addition to these limitations, the models were trained with constant batch size, learning rate, and epochs. Training with different parameters and early stoppage might increase the obtained results. These employments will be considered in further research.

## 6. Conclusions

Ascaris and taeniasis are the most prevalent helminth infections that require rapid and reliable detection and diagnosis. Analyzing samples with egg-based microscopy is time-consuming and might cause misdiagnosis for several reasons. Therefore, advanced tools such as artificial intelligence and deep learning aim to provide reliable diagnosis of Ascaris and taeniasis.

In this study, we considered three advanced deep learning models, EfficientNet V2 S, ConvNeXt Tiny, and MobileNet V3, to evaluate the diagnostic capabilities of new-generation deep learning models in diagnosing ascaris, taeniasis, and uninfected samples. Multi-class experiments were performed, and the results showed that deep learning models could provide robust and accurate diagnostic support to microbiologists and reduce subjectivity. All of the considered models achieved reasonable results; however, the EfficientNet V2 S model obtained superior results and outperformed other models. The results suggest that implementing deep learning models to assist experts would reduce the analysis costs in terms of time and help doctors find appropriate treatment.

To implement these models in clinical settings, validation on a broader range of clinical samples is needed to ensure accuracy across diverse populations. Integration with existing diagnostic systems, along with regulatory approval and training for healthcare professionals, would be essential. Once fully adopted, these AI models could significantly enhance diagnostic efficiency, reducing costs and improving patient outcomes.

Our future work will include considering more diseases and performing laboratory experiments to improve the models’ success and provide an applicable platform.

## Figures and Tables

**Table 1:** Characteristics of Helminth Egg Microscopy Image Collection (HEMIC) Dataset.

Disease	Number of Images	Spatial Dimensions
*Ascaris lumbricoides*	884	3000 × 4000
*Taenia saginata*	440	3000 × 4000
Uninfected	1003	3000 × 4000
Total	2327	-

**Table 2:** All results of multinomial experiments.

	*Ascaris*			*Taenia*			Uninfected		
Model	Precision	Recall	F1-Score	Precision	Recall	F1-Score	Precision	Recall	F1-Score
ConvNeXt Tiny	**0.9793**	0.9743	0.9767	0.9529	0.9744	0.963	0.9819	0.9759	0.9789
EfficientNet V2 S	0.9756	**0.9846**	**0.9801**	**0.9863**	**0.981**	**0.9835**	**0.988**	**0.9818**	**0.9849**
MobileNet V3 S	0.9469	0.9731	0.9596	0.9653	0.9465	0.9557	0.9816	0.9623	0.9716

Bold values indicate the highest scores.

**Table 3:** Macro-averaged results.

Model	Macro Precision	Macro Recall	Macro F1-Score
ConvNeXt Tiny	0.9714	0.9748	0.9729
EfficientNet V2 S	**0.9833**	**0.9825**	**0.9828**
MobileNet V3 S	0.9646	0.9606	0.9623

**Table 4:** The *p*-values of evaluation metrics.

Precision	Recall	F1-Score
0.0559	0.0101	0.0005

**Table 5:** Mean Differences Comparison (Recall).

Group 1	Group 2	Mean Difference	*p*-Adjusted	Lower Bound	Upper Bound
ConvNext Tiny	EfficientNet V2 S	0.0076	0.5198	−0.0092	0.0244
ConvNext Tiny	MobileNet V3 S	−0.0142	0.1113	−0.031	0.0026
EfficientNet V2 S	MobileNet V3 S	−0.0218	0.0081	−0.0386	−0.005

**Table 6:** Mean Differences Comparison (F1-Score).

Group 1	Group 2	Mean Difference	*p*-Adjusted	Lower Bound	Upper Bound
ConvNext Tiny	EfficientNet V2 S	0.0099	0.1089	−0.0017	0.0216
ConvNext Tiny	MobileNet V3 S	−0.0106	0.0826	−0.0223	0.0011
EfficientNet V2 S	MobileNet V3 S	−0.0205	0.0003	−0.0322	−0.0088

## Data Availability

The data presented in this study are available at https://www.kaggle.com/datasets/omid25s/helminth-egg-microscopy-image-collection-hemic/data, accessed on 22 August 2024.
